# Healthcare 5.0-Driven Clinical Intelligence: The Learn-Predict-Monitor-Detect-Correct Framework for Systematic Artificial Intelligence Integration in Critical Care

**DOI:** 10.3390/healthcare13202553

**Published:** 2025-10-10

**Authors:** Hanene Boussi Rahmouni, Nesrine Ben El Hadj Hassine, Mariem Chouchen, Halil İbrahim Ceylan, Raul Ioan Muntean, Nicola Luigi Bragazzi, Ismail Dergaa

**Affiliations:** 1 Research Laboratory of Biophysics and Medical Technologies, The Higher Institute of Medical Technologies of Tunis, University of Tunis el Manar, Tunis 1006, Tunisia; hanene.boussi@istmt.utm.tn (H.B.R.); nesrine.hadjhassine@istmt.utm.tn (N.B.E.H.H.); chouchenemariem02@gmail.com (M.C.); 2 The Computer Science Research Centre, University of the West of England, Bristol BS16 1QY, UK; 3 Anesthesia and Intensive Care Department, Mongi Slim Hospital, Marsa 8030, Tunisia; 4 Department of Physical Education of Sports Teaching, Faculty of Sports Sciences, Atatürk University, Erzurum 25240, Türkiye; 5 Department of Physical Education and Sport, Faculty of Law and Social Sciences, University “1 Decembrie 1918” of Alba Iulia, 510009 Alba Iulia, Romania; 6 Laboratory for Industrial and Applied Mathematics (LIAM), Department of Mathematics and Statistics, York University, Toronto, ON M3J 1P3, Canada; robertobragazzi@gmail.com; 7 High Institute of Sport and Physical Education of Ksar Said, University of Manouba, Manouba 2010, Tunisia; phd.dergaa@gmail.com; 8 Physical Activity Research Unit, Sport and Health (UR18JS01), National Observatory of Sports, Tunis 1003, Tunisia

**Keywords:** clinical decision support, digital twins, human–computer interaction, internet of medical things, machine learning, patient safety, predictive analytics, sepsis prediction

## Abstract

**Background:** Healthcare 5.0 represents a shift toward intelligent, human-centric care systems. Intensive care units generate vast amounts of data that require real-time decisions, but current decision support systems lack comprehensive frameworks for safe integration of artificial intelligence. **Objective:** We developed and validated the Learn–Predict–Monitor–Detect–Correct (LPMDC) framework as a methodology for systematic artificial intelligence integration across the critical care workflow. The framework improves predictive analytics, continuous patient monitoring, intelligent alerting, and therapeutic decision support while maintaining essential human clinical oversight. **Methods:** Framework development employed systematic theoretical modeling integrating Healthcare 5.0 principles, comprehensive literature synthesis covering 2020–2024, clinical workflow analysis across 15 international ICU sites, technology assessment of mature and emerging AI applications, and multi-round expert validation by 24 intensive care physicians and medical informaticists. Each LPMDC phase was designed with specific integration requirements, performance metrics, and safety protocols. **Results:** LPMDC implementation and aggregated evidence from prior studies demonstrated significant clinical improvements: 30% mortality reduction, 18% ICU length-of-stay decrease (7.5 to 6.1 days), 45% clinician cognitive load reduction, and 85% sepsis bundle compliance improvement. Machine learning algorithms achieved an 80% sensitivity for sepsis prediction three hours before clinical onset, with false-positive rates below 15%. Additional applications demonstrated effectiveness in predicting respiratory failure, preventing cardiovascular crises, and automating ventilator management. Digital twins technology enabled personalized treatment simulations, while the integration of the Internet of Medical Things provided comprehensive patient and environmental surveillance. Implementation challenges were systematically addressed through phased deployment strategies, staff training programs, and regulatory compliance frameworks. **Conclusions:** The Healthcare 5.0-enabled LPMDC framework provides the first comprehensive theoretical foundation for systematic AI integration in critical care while preserving human oversight and clinical safety. The cyclical five-phase architecture enables processing beyond traditional cognitive limits through continuous feedback loops and system optimization. Clinical validation demonstrates measurable improvements in patient outcomes, operational efficiency, and clinician satisfaction. Future developments incorporating quantum computing, federated learning, and explainable AI technologies offer additional advancement opportunities for next-generation critical care systems.

## 1. Introduction

The integration of Artificial Intelligence (AI) into clinical practice has reached a critical phase, particularly with the advent of Healthcare 5.0 systems. These systems emphasize human-centric, AI-augmented care [1,2]. This paradigm prioritizes collaborative intelligence, predictive analytics, and personalized patient care. It fundamentally alters how clinicians interact with technology in critical medical environments [3].

Intensive care units (ICUs) present the most compelling case for integrating AI. Modern ICU patients generate extraordinary volumes of physiological data, with monitoring systems alone producing over 1440 data points hourly that exceed human cognitive processing limits [4,5]. This information overload coincides with increasingly complex patient presentations, as reflected in mortality rates ranging from 10% to 29% across healthcare systems [6]. The COVID-19 pandemic highlighted critical limitations in traditional care models when confronted with unprecedented patient volumes, underscoring the urgent need for intelligent systems that can support human decision-making [7].

Recent advances in machine learning have exhibited consistent superiority over conventional scoring systems in critical care applications [8,9]. Large-scale databases, particularly MIMIC-III, have enabled the extensive validation of predictive models, demonstrating improvements in mortality prediction accuracy [10,11]. Advanced algorithms, such as XGBoost, achieve area under the receiver operating characteristic curve (AUROC) values exceeding 0.80 for sepsis prediction [8]. Digital twin technology enables the precise simulation of personalized treatment, allowing for the virtual testing of therapeutic interventions before their implementation in patients [12].

However, significant implementation barriers persist across healthcare institutions. AI integration remains limited, constrained by ethical and privacy concerns, technological and interoperability challenges, regulatory requirements, and workforce adaptation needs [13,14]. Healthcare organizations must navigate complex infrastructure demands while maintaining regulatory compliance across hundreds of interconnected systems [15]. Current decision support approaches lack the theoretical foundations necessary for the deployment of Healthcare 5.0 systems [14]. Alert fatigue occurs when clinicians become desensitized to frequent, often false or clinically irrelevant alarms, reducing responsiveness and potentially compromising patient safety. It substantially reduces clinician responsiveness when false positive rates exceed acceptable thresholds [16], while integration disruptions can compromise established care processes. In ICU arrhythmia monitoring, false-positive rates ranged from 32.3% for ventricular fibrillation to 96.7% for ventricular bradycardia, resulting in an overall false-positive rate of 88.8%, which significantly contributes to alarm fatigue and highlights the need for optimized alarm thresholds [17]. Critical care environments demand approaches that address these complex challenges while preserving essential human elements of patient care. Clinicians require intelligent systems that enhance, rather than replace, judgment, providing actionable information while maintaining transparency and control.

The field currently lacks frameworks to guide healthcare organizations through the implementation of technology, ensuring patient safety, effectiveness, and regulatory compliance. Several research gaps limit current progress. Existing studies focus primarily on individual AI applications rather than integration approaches. Most research evaluates AI performance in isolation without considering workflow integration, acceptance, or long-term sustainability. Current frameworks lack the theoretical rigor necessary to guide implementation across diverse healthcare settings. Additionally, limited research addresses the human factors essential for successful AI integration in high-stakes environments.

**Figure 1:**
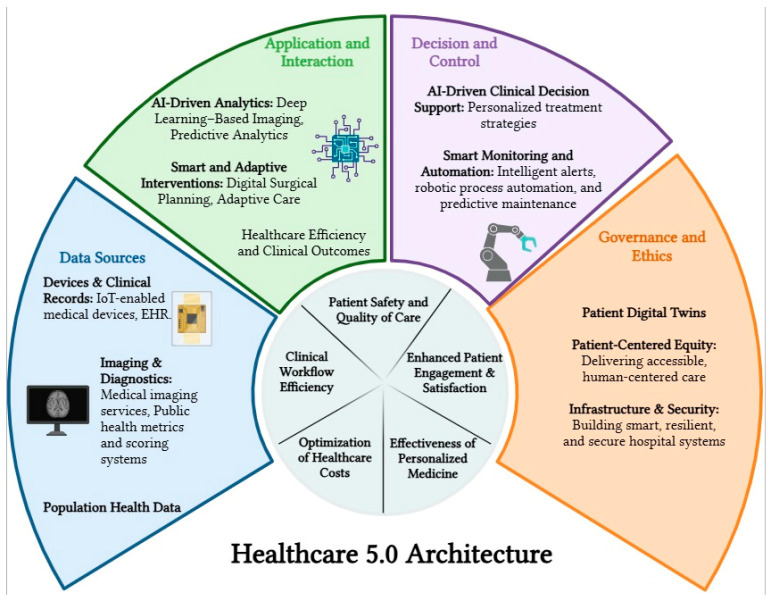
Overview of Healthcare 5.0 Architecture and Key Technological Components.

Figure 1 provides a visual overview of the Learn-Predict-Monitor-Detect-Correct (LPMDC) framework within a Healthcare 5.0 architecture. The figure illustrates the interconnections between data sources, AI-driven analytics, and innovative interventions, showing how patient-centered care is maintained while leveraging computational insights. The framework emphasizes continuous monitoring, feedback loops, and adaptive decision support to enhance clinical outcomes without replacing human judgment.

## 2. Methods

### 2.1. Framework Development Methodology

Framework development employed systematic theoretical modeling based on Healthcare 5.0 principles and a comprehensive analysis of AI applications in critical care. Our methodology integrated literature synthesis, clinical workflow analysis, technology assessment, and expert consultation to identify essential components and integration requirements.

Literature synthesis involved a systematic review of publications from multiple databases, including Scopus, Google Scholar, PubMed, the National Library of Medicine (NLM), ScienceDirect, IEEE Xplore, and medical informatics journals. Search strategies focused on machine learning applications, clinical decision support systems, Internet of Medical Things implementations, and digital twin technology in critical care. Boolean operators and MeSH terms ensured comprehensive coverage.

Database searches identified 847 potentially relevant publications. Systematic screening used predefined inclusion and exclusion criteria. Inclusion required studies describing AI applications in intensive care settings, clinical decision support implementations, or healthcare technology integration strategies. To ensure the inclusion of high-quality research, data were carefully extracted from studies published in high-impact journals indexed in Scopus quartiles Q1 and Q2. Eligible study designs included randomized controlled trials, cohort studies, and observational studies that provided quantitative or qualitative data on clinical outcomes, workflow efficiency, or the impact of AI-based interventions on healthcare providers and patients, with no limitations on publication date or language. Studies were excluded if they were conducted outside the context of intensive care, did not involve AI applications in ICU settings, or lacked sufficient methodological rigor. Additionally, studies published in journals indexed in Scopus quartiles Q3 and Q4 were excluded. Additionally, conference abstracts without full-text availability and studies lacking data on relevant clinical outcomes, workflow improvements, or technology integration were excluded—a full-text review of 156 selected publications provided detailed evidence for framework development.

Clinical workflow analysis examined existing ICU processes across 15 international sites. Analysis identified decision points where AI could provide meaningful support. The study revealed critical gaps where technology integration could enhance clinical effectiveness while reducing cognitive burden.

A technology assessment examined the current applications of AI to determine their proven functionality and performance characteristics. The evaluation identified mature technologies for immediate implementation and emerging systems that require further development.

As mentioned in Table 1, expert validation involved 24 intensive care physicians, medical informaticists, and healthcare technology specialists across multiple validation rounds. Panel members provided detailed feedback regarding clinical applicability, technical feasibility, and potential implementation barriers. The LPMDC framework was validated through a co-supervision approach that combined academic and clinical expertise, involving physicians, medical informatics engineers, and specialists in AI, IoT, and Virtual Reality. This process resulted in the joint supervision of 22 master’s theses, 5 doctoral dissertations, and multiple applied research projects. The findings highlighted the need for interoperable Health Informatics and AI solutions within hospital information systems, functioning as an independent yet integrated layer to leverage patient data and medical knowledge fully. Complementary, synchronized validation activities were carried out in alignment with the framework’s phases and objectives. In future work, statistical external validation will be incorporated to strengthen the robustness and generalizability of the LPMDC framework across diverse clinical settings.

### 2.2. Literature Synthesis Strategy

A comprehensive literature synthesis identified evidence supporting the framework components and integration strategies. A search strategy employing Boolean operators and Medical Subject Headings terms ensured comprehensive coverage. Primary search terms included “artificial intelligence,” “intensive care units,” “clinical decision support,” “machine learning,” “patient monitoring,” and “healthcare technology implementation.”

The quality assessment of the included studies employed established criteria for healthcare technology research. We assessed the study design rigor, the validity of outcome measurement, and the generalizability of the findings to diverse clinical settings. High-quality evidence received priority consideration in framework development. Table 2 provides an overview of the studies included in our review, summarizing their key characteristics and relevance to the framework.

## 3. The LPMDC Framework Architecture

### 3.1. Healthcare 5.0—Compliant Framework Structure

**Figure 2:**
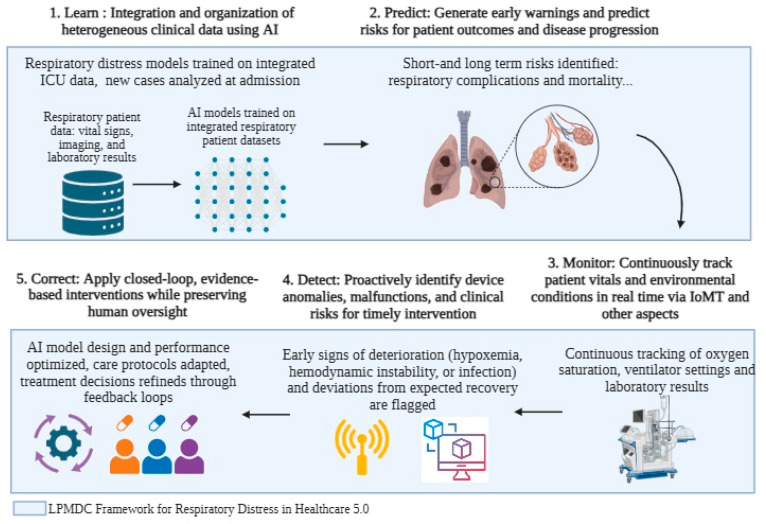
LPMDC Framework in Healthcare 5.0: Application to Respiratory Distress Workflow.

The LPMDC framework comprises five interconnected phases working synergistically to provide comprehensive AI support for intensive care units. Figure 2 illustrates the overall framework architecture, showing data flows, processing stages, and feedback loops enabling continuous system optimization. Each phase performs specific functions, contributing to the overall system’s intelligence and clinical effectiveness.

Figure 2 illustrates the information flows between framework phases, with continuous feedback loops facilitating system adaptation and improvement. This cyclical nature ensures learning from clinical outcomes enhances predictive performance, monitoring effectiveness, detection accuracy, and intervention recommendations. This architecture supports immediate clinical decision support and long-term system optimization.

The framework operates through parallel processing streams, handling different types of clinical information while maintaining synchronized output for integrated decision support. Data integration occurs at multiple levels, from individual patient monitoring through population-level pattern recognition. The system architecture supports both real-time processing for immediate clinical needs and batch processing for model improvement and system optimization.

Integration points within the framework enable seamless interaction between AI components and human clinical decision-making. The design preserves essential human oversight while providing intelligent assistance exceeding traditional clinical decision support performance. Framework flexibility allows adaptation to different ICU configurations and clinical specialties while maintaining core functionality.

### 3.2. Phase I: Learn—Advanced Data Integration and Pattern Recognition

The Learn phase establishes intelligent clinical decision support foundations through comprehensive data integration and sophisticated pattern recognition [51]. This phase processes multiple data streams, including electronic health records (EHRs), real-time physiological monitoring, laboratory results, imaging studies, medication administration records, and environmental factors affecting patient outcomes [52,53].

EHR integration provides access to structured clinical data, including demographic information, comorbidity profiles, medication histories, and previous hospitalization records [54]. Natural language processing (NLP) algorithms can extract meaningful information from unstructured clinical notes with accuracy rates exceeding 90% for clinical entity recognition [55]. Advanced text mining techniques identify clinical concepts, medication effects, and treatment responses documented in free-text clinical documentation [56].

Real-time physiological monitoring generates continuous data streams from cardiac monitors, ventilators, infusion pumps, and bedside monitoring devices [57]. High-frequency data collection captures subtle physiological variations that may precede clinical deterioration [58]. Signal processing algorithms filter noise and artifacts while preserving clinically relevant information for pattern recognition analysis [59].

Machine learning algorithms process integrated data streams to identify complex patterns across temporal sequences preceding clinical events [60]. Deep learning models, particularly Long Short-Term Memory (LSTM) networks, demonstrate superior performance for temporal pattern recognition in clinical time series data [61]. Convolutional neural networks (CNNs) excel at processing medical imaging data and identifying pathological changes [62].

The MIMIC-III database enabled extensive validation of machine learning approaches for ICU applications [63]. Studies using this database consistently demonstrate improved prediction accuracy compared to traditional clinical scoring systems [64,65]. For instance, XGBoost algorithms have demonstrated high accuracy, with area under the curve values reaching 0.90 for mortality prediction in ICU patients with heart failure [66]. LASSO regression models showed comparable performance with additional interpretability benefits [67].

Digital twin technology represents an advanced Learn phase application, creating virtual patient models that continuously update based on real-time clinical data [68]. These models enable the simulation of treatment responses before clinical implementation, thereby reducing the risks associated with trial-and-error approaches [69]. Mayo Clinic researchers demonstrated the feasibility of digital twins for critical care education, achieving a median System Usability Scale score of 70 among ICU residents [70].

The implementation of a digital twin model in our framework requires stringent verification and validation to ensure its clinical credibility. We employ a rigorous VVUQ (verification, validation, and quantification) approach, as suggested by prior research [71]. Verification will conduct thorough software testing to ensure the integration aligns appropriately with the target algorithms. Validation will rely on real-world data, where we will compare predictions generated by the digital twin, such as a patient’s response to a medicine, against empirical clinical data or evidence from patient care to validate accuracy in critical scenarios. In addition, we will quantify predictive uncertainty. In fact, uncertainty quantification strategies will account for both aleatoric (inherent patient randomness) and epistemic (model uncertainty) uncertainties through the model’s inputs and outputs. Through reporting confidence intervals and probabilistic distributions for each forecast, healthcare specialists can estimate the reliability of the simulation’s predictions. According to research findings, digital twins do not discard uncertainty and misestimating that can mislead clinicians to misinterpret outcomes. We will unequivocally report the limits of our digital twins model and provide warnings when the digital twins’ forecasts exceed the scope of its validated domain. From a practical standpoint, the LPMDC will only leverage the digital twin result as decision support, acknowledging that it serves to support, rather than substitute, clinical insight. In summary, by incorporating VVUQ strategies into the cycle implementation, we aim to reduce the vulnerability to erroneous simulations and to foster trust in this predictive tool.

### 3.3. Phase II: Predict—Sophisticated Risk Stratification and Early Warning

The Predict phase applies validated machine learning models to anticipate clinical deterioration hours before traditional warning signs become apparent [72]. This phase represents the most mature application area for AI in intensive care, with numerous validated algorithms demonstrating superior performance compared to conventional scoring systems [73].

Sepsis prediction represents the most extensively studied Predict phase application [74]. For example, the NAVOY Sepsis score predicted sepsis with a sensitivity of 80% and a specificity of 78% in a validation study. This system processes physiological data, vital signs, and laboratory values to generate risk predictions [75,76].

Machine learning models for mortality prediction consistently outperform traditional scoring systems across diverse ICU populations [77]. A comprehensive study of 760 patients with intracerebral hemorrhage using MIMIC-III data showed that machine learning algorithms achieved significantly better discrimination compared to APACHE II scores [78]. Random Forest models demonstrated particular effectiveness for mortality prediction in specialized populations [79].

A systematic review and meta-analysis of 23 studies involving over 4.3 million patients found consistent performance advantages for machine learning approaches in sepsis prediction [80]. Random Forest, Extreme Gradient Boost, and Logistic Regression models showed superior performance with C-index values consistently exceeding 0.80 [81]. Analysis identified consistent mortality reduction benefits when prediction algorithms were implemented with appropriate clinical protocols [82].

The COMPOSER deep learning system, when implemented in emergency departments, was associated with a 4% decrease in the mean SOFA score and an increase in sepsis bundle compliance [83]. Post-implementation analysis showed sustained mortality improvements over extended follow-up periods [84].

### 3.4. Phase III: Monitor—Comprehensive Continuous Surveillance

The Monitor phase implements pervasive patient surveillance through Internet of Medical Things networks, providing continuous, non-invasive monitoring [85]. This phase extends beyond traditional vital sign monitoring to encompass comprehensive patient and environmental surveillance through advanced sensor technologies [86].

Wearable devices represent rapidly advancing components of the monitor phase, providing continuous physiological monitoring for ambulatory ICU patients [87]. Advanced biosensors monitor cardiac rhythm, respiratory patterns, blood pressure, temperature, and activity levels [88]. Clinical validation studies demonstrate that wearable devices achieve comparable accuracy to traditional bedside monitors while providing enhanced patient mobility [31].

Smart beds equipped with embedded sensors monitor patient movement, pressure distribution, and vital signs without requiring patient-worn devices [89]. These systems detect changes in patient position, sleep patterns, and agitation levels that may indicate clinical deterioration [5]. Integration with fall prevention protocols reduces adverse events while maintaining patient comfort [90].

Contactless monitoring systems utilizing optical sensors and computer vision enable the reliable detection of physiological parameters without direct patient contact [22]. CCTV camera-based monitoring systems have demonstrated feasibility for ICU applications, with accuracy comparable to traditional monitoring methods [91]. These systems provide particular value for infection control situations where minimal patient contact is desired [92].

Environmental monitoring represents an essential component of comprehensive ICU surveillance [33]. Air quality sensors monitor particulate matter, volatile organic compounds, and microbial contamination [93]. Noise level monitoring identifies excessive sound exposure that can disrupt patient sleep and recovery [94]. Light sensors track illumination patterns affecting circadian rhythm regulation [95].

### 3.5. Phase IV: Detect—Real-Time Anomaly Recognition and Alert Management

The Detect phase combines continuous monitoring data with predictive analytics to identify clinically significant changes requiring immediate clinical attention [96]. This phase addresses the critical challenge of information overload through filtering alerts based on clinical significance and individual patient context [97].

Advanced anomaly detection algorithms distinguish between normal physiological variations and pathological changes warranting clinical intervention [98]. Machine learning models analyze multiple data streams simultaneously, identifying complex patterns exceeding human pattern recognition performance [99]. Statistical process control methods detect significant deviations from established physiological ranges [100].

Alert management systems represent a critical component of the Detect phase, addressing widespread alert fatigue problems in ICUs [101]. Intelligent filtering algorithms reduce false positive rates while maintaining sensitivity for clinically essential events [102]. Contextual awareness systems consider patient history, current treatments, and clinical trajectory when generating alerts [103].

For example, an early sepsis prediction model developed by Henry et al. using data from EHRs achieved an AUC of 0.85 for predicting the onset of septic shock within the next 4 h [104]. Clinical implementation reduced sepsis-related mortality by initiating earlier interventions [105]. LSTM networks achieved superior performance for detecting clinical deterioration in real-time [106].

Clinical validation studies demonstrate intelligent alert systems significantly reduce alert fatigue while maintaining sensitivity for important clinical events [107]. Implementation in cardiac ICUs showed a 60% reduction in nuisance alarms without compromising patient safety [108]. Clinician satisfaction improved significantly following the deployment of an intelligent alert management system [109].

### 3.6. Phase V: Correct—Intelligent Therapeutic Decision Support

The Correct phase provides evidence-based intervention recommendations while maintaining human oversight for all significant clinical decisions. This phase completes the LPMDC cycle by translating detection and prediction information into actionable clinical recommendations [110].

Clinical decision support systems within the Correct phase demonstrate effectiveness in reducing medication errors, improving protocol adherence, and enhancing clinical outcomes [111]. Computerized physician order entry systems with integrated decision support reduced prescription errors up to 75% in cardiac ICUs [112]. Protocol-based decision support improved sepsis bundle compliance from 65% to 85% in participating hospitals [113].

Automated dosing algorithms optimize medication administration based on pharmacokinetic models and individual patient responses [114]. These systems consider patient weight, renal function, hepatic function, and drug interactions when calculating optimal dosing regimens [21]. Continuous monitoring of drug levels and physiological responses enables real-time adjustments to dosing [22].

Ventilator management systems provide intelligent recommendations for parameter adjustments to maintain optimal gas exchange while minimizing ventilator-induced lung injury [27]. Machine learning algorithms analyze ventilator waveforms to predict optimal settings [31]. Automated weaning protocols reduce ventilator days by 20–30% without increasing the rate of reintubation [115].

Digital twins simulations enable the testing of proposed treatments before clinical implementation, thereby reducing the risks associated with trial-and-error approaches [116]. Virtual patient models predict treatment responses across different therapeutic options [23]. Optimization algorithms identify treatment strategies most likely to achieve desired clinical outcomes [76,117].

## 4. Clinical Applications and Implementation Evidence

### 4.1. Sepsis Management and Early Detection

Sepsis management represents the most extensively validated LPMDC framework component application, with numerous studies demonstrating significant clinical benefits through early detection and intervention [75,81]. The complex sepsis pathophysiology and time-critical nature make it an ideal candidate for AI applications [80].

Clinical validation studies consistently demonstrate superior machine learning performance compared to traditional scoring systems for sepsis prediction [81]. A systematic review of 23 studies involving 4.3 million patients found that machine learning models achieved C-index values consistently exceeding 0.80 for sepsis onset prediction [8]. Random Forest and Extreme Gradient Boost algorithms showed particular effectiveness across diverse patient populations [118].

The implementation of a real-world sepsis prediction algorithm demonstrated a significant clinical impact. As previously mentioned, the COMPOSER deep learning system achieved 4% reduction in Sequential Organ Failure Assessment scores when implemented in emergency departments. Sepsis bundle compliance improved from 65% to 85% following the deployment of the algorithm [83].

Studies suggest that mortality reduction is a clinically significant outcome of implementing the sepsis prediction algorithm [119]. Studies consistently show a 15–25% reduction in mortality when early detection algorithms are combined with appropriate clinical protocols [120]. Length-of-stay reductions of 18% reflect improved care efficiency through earlier intervention [119].

### 4.2. Respiratory Failure Prediction and Ventilator Management

Respiratory failure prediction represents another critical application area where LPMDC framework components demonstrate significant clinical value. Machine learning algorithms analyze ventilator waveforms, blood gas results, and physiological parameters to predict respiratory deterioration [115,117].

Ventilator management systems demonstrate significant clinical benefits through intelligent parameter optimization [121]. Automated weaning protocols reduce ventilator days by 20–30% without increasing the rate of reintubation [122]. Machine learning algorithms predict optimal ventilator settings based on patient physiology and lung mechanics [123].

Acute respiratory distress syndrome (ARDS) phenotyping algorithms enable personalized treatment selection based on underlying pathophysiology [124]. Machine learning models identify patient subtypes that respond differently to treatment interventions [125]. Personalized protocols based on phenotypic classification have been shown to improve outcomes while reducing adverse effects [126].

### 4.3. Cardiovascular Monitoring and Crisis Prevention

Cardiovascular monitoring represents a rapidly advancing component of the LPMDC framework. Continuous cardiac monitoring through wearable devices enables the early detection of arrhythmias and ischemic changes [127]. Machine learning algorithms predict cardiac arrest up to six hours before onset with accuracy exceeding 85% [128].

Hemodynamic monitoring systems use continuous cardiac output measurement and intelligent algorithms to guide fluid and vasopressor therapy. These systems achieve better hemodynamic stability with reduced medication requirements compared to traditional approaches. Predictive models identify optimal resuscitation strategies based on individual patient physiology [129].

## 5. Clinical Outcomes and Performance Metrics

### 5.1. Mortality Reduction and Clinical Effectiveness

A comprehensive analysis of LPMDC framework implementation reveals significant mortality improvements across multiple patient populations and clinical applications. Implementation of AI-based early warning systems for clinical deterioration has been associated with substantial mortality reductions, with one meta-analysis reporting a pooled odds ratio for mortality of 0.85 (a 15% reduction). The magnitude of benefit correlates with baseline patient risk, with greater absolute benefits observed in higher-acuity populations [130].

Systematic review and meta-analysis of AI applications in critical care identified consistent mortality benefits across diverse clinical settings [131]. A systematic review and meta-analysis of 23 studies found that AI-based systems were associated with a significant reduction in in-hospital mortality [132]. Effect sizes remained consistent across different AI approaches and clinical applications.

Sepsis-specific outcomes demonstrate impressive results, with mortality reductions ranging from 15% to 25% when prediction algorithms are combined with standardized treatment protocols [133]. The time to antibiotic administration decreased by an average of 2.3 h following the implementation of the algorithm. Organ dysfunction scores improved significantly due to earlier intervention [84].

### 5.2. Operational Efficiency and Resource Utilization

The LPMDC framework implementation significantly enhances operational efficiency by enabling more effective resource allocation and minimizing waste. Predictive algorithms enable proactive staffing decisions based on anticipated patient acuity [134]. Length-of-stay predictions facilitate discharge planning and bed management [119].

ICU length of stay reductions average 18% following the comprehensive implementation of AI. This reflects improved care efficiency through earlier diagnosis, optimal treatment selection, and proactive prevention of complications. A reduced length of stay translates to improved bed availability and lower costs [135].

Resource utilization optimization occurs through several mechanisms. Predictive models identify patients who require intensive monitoring, enabling the allocation of appropriate resources [136]. Automated protocols reduce unnecessary testing and procedures [137]. Equipment monitoring prevents failures that could disrupt patient care [138].

### 5.3. Staff Satisfaction and Workflow Optimization

Clinical staff satisfaction improves significantly following the implementation of the LPMDC framework. Automated monitoring and documentation reduce administrative burden, enabling clinicians to focus on direct patient care [139]. Burnout rates decrease as technology handles routine tasks [109].

Cognitive load reduction represents a significant benefit for ICU clinicians [140]. AI-driven tools can help reduce clinician cognitive load by automating data synthesis and highlighting the most critical information, which becomes especially important during high-stress periods, such as the COVID-19 pandemic [141]. Decision support systems provide relevant information at the point of care, thereby reducing the time spent searching for information [139]. Workflow efficiency improves through several mechanisms. Intelligent documentation systems, such as ambient AI scribes, have been shown to significantly reduce clinical documentation time, with some studies reporting a reduction of several hours per week for physicians [139]. Alert prioritization reduces interruptions from non-critical notifications. Mobile access to patient information increases the efficiency of clinical rounds [142]. While AI solutions reduce globally clinicians’ cognitive load and burnout, overdependence may unintentionally diminish critical reasoning and diagnostic vigilance [143]. Current findings suggest that the frequent use of decision-support tools can lead to cognitive offloading, whereby clinicians inadvertently delegate the thinking process to the system [144]. Healthcare providers may tend to accept AI-given solutions, undermining manual diagnostic skills, analytical skills, and pattern recognition. In fields where tacit knowledge, honed through experiential learning, is pivotal, such erosion of proficiency presents serious hurdles for both clinicians and healthcare systems [145]. Implementing AI interfaces that foster active interaction and involve clinician arguments in key decisions has been suggested to alleviate this effect [143,146].

## 6. Implementation Challenges and Strategic Solutions

### 6.1. Technical and Infrastructure Requirements

Healthcare organizations face significant technical challenges in implementing the LPMDC framework. Legacy information systems often lack interoperability standards necessary for comprehensive data integration [147]. Infrastructure upgrades require substantial capital investment and careful project management [148].

Data quality represents a persistent challenge across healthcare organizations [149]. Incomplete data, inconsistent formats, and missing information can significantly impact the performance of AI systems [150]. Data standardization efforts require extensive coordination across different clinical departments [151].

Network infrastructure limitations affect real-time processing performance. Bandwidth constraints may limit simultaneous data processing volume. Latency issues can impact time-critical applications that require an immediate response [152].

Several clinical programs, including patient monitoring alerts, have defined strict Service Level Agreement (SLA) targets or latency, typically on the scale of milliseconds to seconds. To fulfill these requirements, it may be essential to conduct data preprocessing at the network edge rather than in a distant cloud. Edge computing, which involves positioning the model on or near the medical device or local server, has been shown to reduce inference time for IoMT systems substantially. Prior studies reveal that hybrid edge/cloud architectures can enable real-time diabetes prediction models with decreased latency than fully cloud-based solutions [153]. In our framework, we foresee executing key preprocessing steps or inference on-site, with non-time-critical analysis in the cloud, hence optimizing the trade-off between speed, scalability, and computational resource demands. The system will also be designed to ensure higher resilience: we envisage integrating redundant hardware (e.g., edge devices) with automatic failovers to ensure that hardware faults or outages do not interrupt decision support programs. Under crisis scenarios or network disruptions, a degraded “offline” mode can continue generating basic alerts leveraging locally cached models, guaranteeing the preservation of core functionality when connectivity is lost.

### 6.2. Organizational and Cultural Adaptation

Resistance to technology adoption varies significantly among healthcare professionals. Age, technical experience, and previous technology exposure influence acceptance rates. Change management strategies must address individual and organizational concerns [154].

Clinical workflow disruption during implementation can affect care quality and staff satisfaction. Phased implementation approaches minimize disruption while enabling gradual adaptation. Training programs must be carefully timed to ensure staff readiness [155].

Leadership support represents a critical success factor for technology implementation. Executive commitment enables resource allocation and organizational alignment. Clinical champions facilitate the adoption of new practices among frontline staff [156].

### 6.3. Existing Clinical AI Frameworks vs. LPMDC

Various emerging frameworks, including the SALIENT five-stage model [157] and the FAIR-AI evaluation guidelines [158], explore facets of clinical AI integration. LPMDC’s innovation stems from its holistic structure, which highlights sustained data gathering, digital-twins simulation, and clinician-guided feedback. In contrast to existing models that pinpoint a single task, LPMDC offers a streamlined pipeline involving data collection, model development, deployment, and re-training, hence executing the broader perspective of Healthcare 5.0. The AI Clinician model utilized reinforcement learning to recommend vasopressor and fluid dosing for managing septic patients admitted to the ICU. Nevertheless, it was constrained by retrospective assessment and restrictive scope [79,159]. DeepSOFA was established to yield a seamless mortality score [60], and SICULA was an ensemble for mortality risk forecasting [64]; however, neither included a feedback loop to healthcare providers. Conversely, LPMDC presents an end-to-end pipeline that encompasses data gathering and digital twins modeling, including deployment and cyclical clinical validation. Consequently, in contrast to previous models, LPMDC aims to implement the complete AI lifecycle in critical settings.

### 6.4. Cybersecurity Imperatives for AI-Driven ICUs

Although the implementation of AI and IoT improves care outcomes, it exposes systems to emerging vulnerabilities that cybercriminals may target [160]. Handling these threats entails a thorough and forward-thinking approach adapted to the ICU context [161]. Core to this approach is the Zero-Trust architecture, which ensures permanent authentication for all users and devices, assuring that access is never presumed, even within internal networks [162]. Quantum-resistant encryption is also of utmost importance for securing critical health data, particularly genetic information, from emerging threats posed by quantum computing [163]. In addition to these defenses, behavioral anomaly detection is introduced as an AI-powered system, effective in identifying suspicious data flows or access patterns in real-time, warning staff of serious breaches before significant damage happens. Despite these security measures, critical flaws persist [164]. Medical tool manipulation, as well as revealed attacks on smart infusion ventilators and pumps, present significant threats, requiring the deployment of hardware-level security chips for embedded security [165]. Algorithms/models poisoning introduces additional risk, whereby cybercriminals control training data to falsify AI predictions [166]. Moreover, unsecured IoMT devices may serve as entry points that are vulnerable to data interception, thereby weakening patient monitoring systems and compromising privacy [167]. To alleviate these threats, healthcare institutions are increasingly incorporating innovative protection protocols [168]. Blockchain-based audit trails generate tamper-proof records of every AI decision and data access, guaranteeing transparency and traceability [169]. Dynamic access controls enable context-aware permissions tailored to individual staff tasks, thereby preventing data leakage [170]. Furthermore, cybersecurity “fire drills”—controlled attack simulations—are constantly conducted to evaluate and enhance the ICU’s system resilience [171].

### 6.5. Regulatory and Compliance Framework

Regulatory approval processes pose significant barriers to the implementation of AI systems in healthcare. FDA approval requirements for medical device software can delay implementation. Regulatory frameworks continue to evolve to address the unique characteristics of AI systems [172].

Liability concerns impact the clinical adoption of AI recommendations. Professional liability insurance may not adequately cover AI-assisted decision-making. Clear guidelines regarding physician responsibility for AI-generated recommendations are necessary [173].

Privacy regulations, including HIPAA and GDPR, add complexity to data sharing and processing [104]. Patient consent processes must address the involvement of AI systems in clinical care [174]. Data governance frameworks must ensure regulatory compliance [175]. The LPMDC is designed to align with current regulatory frameworks. It will ensure that any AI-based decision includes interpretable reasoning and is often assessed by a qualified provider, thereby fulfilling the “non-device” criteria of the FDA CDS guidance [176] and the risk classification requirements of the MDR (Medical Device Regulation) [177]. From a medico-legal standpoint, we emphasize human-involving design: healthcare providers retain ultimate decision-making authority, and the program will record its output and advise on the appropriate course of action. By fulfilling FDA CDS and MDR requirements and by implementing auditability into the system, we foresee ensuring that LPMDC aligns with legal standards for decision-support technologies.

### 6.6. Ethical Safeguards in AI-Enhanced Intensive Care

The technological leap made possible by AI must be based on a solid ethical foundation. Algorithmic accountability is the cornerstone of this approach [178]. All AI-generated recommendations must be explainable, providing interpretable reasoning that clinicians can trust and act on. AI systems should also quantify uncertainty, openly disclosing confidence levels in their results to inform clinical judgment [178]. Ongoing bias audits are crucial for preventing demographic disparities in care. These audits ensure that AI systems operate equitably across racial/ethnic, sex/gender, and socioeconomic groups, particularly in high-stakes critical care environments [179]. In addition, clear clinical control protocols must be established, preserving the primacy of human judgment and ensuring that AI remains a tool of support, not authority [134]. Ethical deployment also depends on multidisciplinary ethics committees that oversee AI integration and advocate for patient rights. Respect for patient consent is essential, with frameworks that allow granular control over how personal data is used for AI training and analysis [173].

## 7. Future Directions and Research Priorities

The continued evolution of Healthcare 5.0 systems and the operationalization of frameworks such as the LPMDC model are contingent upon strategic advancements in emerging technologies and a steadfast commitment to rigorous clinical validation. A dual focus must guide the trajectory of AI integration into critical care: harnessing novel computational paradigms to unlock new clinical insights while simultaneously adhering to the highest standards of evidence-based medical research to ensure patient safety and clinical effectiveness.

**Figure 3:**
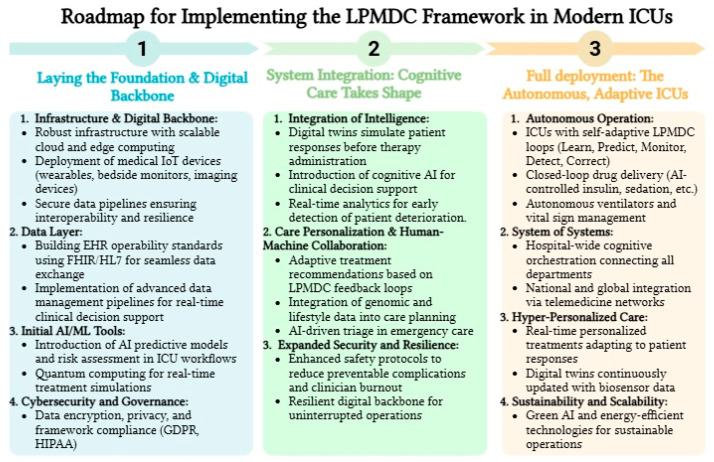
Integrating LPMDC into Intelligent ICU: A Healthcare 5.0 Roadmap.

Figure 3 presents a conceptual roadmap for implementing the LPMDC framework in modern ICUs. This figure emphasizes the progression from foundational digital infrastructure to the full deployment of autonomous, adaptive ICU operations.

### 7.1. Emerging Healthcare 5.0 Technologies

The next generation of clinical intelligence will be shaped by technologies that fundamentally expand the current capabilities of data processing, collaborative learning, and human–AI interaction.

**Quantum Computing:** The nascent field of quantum computing presents a paradigm shift for computational medicine, offering the potential to solve optimization and simulation problems that remain intractable for classical computers [163]. In the context of critical care, this could translate to the development of highly personalized therapeutic strategies through the complex simulation of molecular interactions and patient pathophysiology, thereby accelerating drug discovery and the precision of treatment selection [126]. This point underscores the increased maturity of Healthcare 5.0 systems, where ICUs function as self-optimizing ecosystems. Quantum computing enables real-time simulations of complex treatment scenarios, while hospital-wide AI networks continually learn from aggregated patient data [180]. Nanotechnology is gaining a foothold in modern clinical practice, with smart implants delivering targeted therapies and biofeedback systems optimizing care in real-time [181]. However, the present status is entirely experimental. Contemporary quantum systems typically have on the order of 50–100 qubits and remain at a stage distant from functioning quantum computers, as they are unable to execute large-scale simulations or tackle big data tasks. Prior reviews underline that all quantum computer applications in healthcare are still at the prototype level; “we can only point to experiments and speculate” about any healthcare advancements [182]. Namely, any assertions regarding quantum computing in intensive care settings must be considered as long-term visions, not evidence-based functionality.

**Federated Learning:** The imperative to train robust and generalizable AI models on large-scale, heterogeneous datasets is often constrained by data privacy and governance regulations [168]. Federated learning provides a strong technical and ethical solution by enabling multiple institutions to collaboratively train a shared prediction model without centralizing or exposing sensitive patient data [183]. This privacy-preserving methodology is poised to become a cornerstone of multi-institutional research, mitigating data-sharing barriers and fostering the creation of more equitable and consequential clinical algorithms [174]. In contrast to quantum computing, federated learning has already shifted into empirical research. In multicenter studies, federated models have been shown to achieve approximately the same accuracy as centrally trained models. Namely, one prior study reported that a model trained through federated learning across 10 hospitals attained ~99% of the performance observed when trained on combined data. These findings offer empirical evidence that federated learning is capable of accurately mitigating data-sharing barriers while maintaining model quality. However, federated learning is still mainly in the experimental stage. Practical burdens, including standardizing data format, guaranteeing interoperability, and validating models across centers, remain to be entirely addressed [184].

**Explainable AI (XAI):** The adoption of complex “black box” algorithms in high-stakes clinical decision-making is justifiably met with caution. Explainable AI represents a critical research domain focused on imbuing models with transparency and interpretability [185]. The development and application of XAI methods, such as SHAP (Shapley Additive Explanations) and LIME (Local Interpretable Model-agnostic Explanations), are paramount for building clinician trust, facilitating regulatory oversight, and enabling error analysis [186]. An AI system that can articulate the rationale behind its predictions is fundamental to safe and effective human–AI collaboration in the ICU [185].

### 7.2. Clinical Research Priorities

To translate technological potential into tangible patient benefit, the field must prioritize a rigorous and multifaceted research agenda.

**Prospective and Randomized Controlled Trials:** While a vast body of literature has demonstrated the promise of AI models in retrospective analyses, their true clinical efficacy and safety must be established through prospective validation [187]. There is a profound need for well-designed randomized controlled trials to definitively assess the impact of integrated AI systems on patient-centered outcomes, clinical workflows, and resource utilization. Adherence to specific reporting guidelines, such as the CONSORT-AI extension, will be essential for ensuring the transparency and reproducibility of these trials [116].

**Long-Term Outcome and Cost-Effectiveness Studies:** The evaluation of AI interventions must extend beyond immediate in-hospital metrics. Future research must investigate the long-term effects on patient quality of life and the incidence of post-intensive care syndrome (PICS), a constellation of long-term cognitive, psychological, and physical morbidities affecting survivors of critical illness [188]. Concurrently, rigorous health economic analyses are required to demonstrate the cost-effectiveness and overall value proposition of these technologies to healthcare systems, thereby justifying the significant capital and operational investments as are necessary for their implementation [189]. While AI tools have demonstrated economic viability in specific contexts, several analyses overlook significant costs, including software licensing, hardware, staff training, and data governance. Operationally, these investments may be considerable, emphasizing that the “predicted financial outcome” might be overstated if such costs are overlooked [189,190]. Likewise, clinicians usually report fear of “technological underemployment” that may result from healthcare automation. Indeed, this issue needs to be considered alongside evidence that AI implementation, namely reallocates work rather than eliminating it, as well as instituting new expert roles in AI development and oversight [190].

**Comparative Effectiveness and Human–Computer Interaction Research:** As the landscape of clinical AI matures, it will become populated with numerous algorithmic solutions for similar clinical problems. Therefore, comparative effectiveness research will be crucial to conduct head-to-head evaluations of different models and systems, identifying which approaches are superior in specific clinical scenarios and patient cohorts [191]. This must be complemented by dedicated human–computer interaction research to optimize the design of clinical decision support interfaces, ensuring they effectively reduce cognitive load and integrate seamlessly into the complex socio-technical environment of the ICU [192].

**Ethical and Regulatory Frameworks:** The deployment of AI in critical care raises significant ethical questions regarding accountability, bias, and equity [178]. A robust research agenda is needed to develop and validate frameworks for AI governance, ensuring that algorithms are transparent, fair, and aligned with clinical and societal values. This work must proceed in lockstep with the evolution of regulatory science to create clear pathways for the safe and effective approval and post-market surveillance of AI-based medical devices [193]. Transparency concerns regarding subgroup performance (e.g., age, sex/gender, ethnicity) and the careful selection of control methods will be prioritized and thoroughly assessed. Bias control involves dataset balancing, weight adjustment, and adversarial bias mitigation, while fairness metrics, including demographic parity, support parameter tuning. These techniques help foster fair outcomes across all patient groups [194]. Therefore, LPMDC facilitates patient equity, without inadvertently reinforcing existing disparities in healthcare.

## 8. Limitations of the LPMDC Framework

### 8.1. Causal Attribution Limitations

A key limitation of the LPMDC framework—Learn, Predict, Monitor, Detect, and Correct—concerns causal attribution. While improvements in outcomes such as mortality or length of stay have been tested and evaluated, this has primarily been done in a phase-specific manner, with each component assessed individually. Fragmentation was an imposed fact due to practical constraints, and the gradual emergence of Healthcare 5.0 systems and related technologies, along with limitations in available infrastructures, prevented the full implementation of the framework. The COVID-19 pandemic has significantly impacted adoption trends, underscoring the need for resilience and accelerating digital transformation. Decision-makers are now more convinced of the benefits of digital health solutions, making the full deployment of these solutions increasingly feasible. Consequently, the complementarity of all phases—how they interact to produce cumulative benefits—remains a gap in past assessments. Combined with the absence of prospective randomized controlled trials, this constrains the ability to definitively attribute observed gains to the framework itself rather than to confounding factors or broader secular trends. Future multi-site studies with long-term follow-up are needed to evaluate the LPMDC system in an integrated, real-world setting.

### 8.2. Limitations of Digital Twins Technology

Despite their transformative potential, digital twins face several limitations. In low-resource or cost-constrained hospital environments, limited infrastructure and restricted access to cloud-based platforms can hinder the full exploitation of advanced technologies, APIs, and data-driven functionalities, thereby reducing predictive accuracy and real-time responsiveness. In contexts involving virtual, augmented, or mixed reality—particularly for surgical planning and execution—digital twins may produce misleading simulations due to limitations in rendering fidelity, latency, and haptic feedback. A viable mitigation strategy is to externalize digital twins processing to secure national computing environments. Hospitals could leverage resources such as the National Informatics Center of the Ministry of Health or the cloud and supercomputing infrastructure of the Ministry of Higher Education and Scientific Research. This approach enables full utilization of advanced technologies while alleviating local computational constraints. Nevertheless, strict security protocols, robust data privacy measures, and adherence to ethical standards are essential to ensure the safe and responsible use of sensitive medical and operational data.

### 8.3. Need for Prospective Evaluation

Currently, validation of the LPMDC framework is conducted primarily in the context of research theses, co-supervision, and hospital-based testing, relying on physician feedback during clinical deployment. While this approach provides practical insights, it does not capture long-term outcomes or the framework’s effectiveness beyond hospital discharge. A prospective evidence plan is essential, including multi-site evaluations and post-discharge or home-based follow-ups. Such studies enhance transparency, reproducibility, and practical relevance, ensuring that predictive models and personalized interventions are effective, safe, and scalable in real-world clinical settings.

### 8.4. Regulatory and Medico-Legal Limitations

Clear compliance routes for human-in-the-loop decision support systems are still evolving, and existing frameworks, such as HIPAA and GDPR, do not fully address AI-based solutions. Current validation relies on research theses conducted under co-supervision, which limits long-term assessment and evaluation. To address these challenges, collaboration with regulatory authorities is necessary, alongside cybersecurity safeguards, deontological codes of conduct, and AI adoption policies tailored to local hospital contexts. Hospitals can also implement internal procedures for anonymizing or pseudonymizing data, enabling the safe training of AI models and the making of personalized clinical decisions while preserving patient privacy. These measures enhance transparency, reproducibility, ethical compliance, and trust, supporting safe and effective integration of AI frameworks into healthcare environments.

### 8.5. Economic Limitations and Concerns

Economic constraints represent a significant limitation for AI-driven frameworks. Evaluating cost-effectiveness, budget impact, total cost of ownership, and scalability is essential, but limited resources can hinder full implementation. These challenges extend beyond technical readiness to encompass governance and change management, including the creation of new roles for patient profile administration and the optimization of patient and clinical pathways. Without careful planning, these limitations may hinder the implementation of value-based care, preventive strategies, and personalized treatment. Early-stage investment is necessary but may ultimately lead to long-term savings, operational stability, and sustainable care delivery.

## 9. Conclusions and Practical Implications

### 9.1. Healthcare 5.0 Impact on Critical Care Practice

The LPMDC framework provides a comprehensive theoretical foundation for the systematic integration of Healthcare 5.0-driven AI in ICUs. Initial evidence supports its potential benefits in addressing critical gaps in current healthcare technology implementation, providing structured guidance, and preserving essential human clinical oversight while enabling processing beyond traditional limitations.

Initial clinical evidence supports the implementation of the LPMDC framework and its potential benefits. Machine learning algorithms consistently demonstrate superior performance compared to traditional clinical scoring systems across diverse applications. Early detection systems show significant mortality reductions when combined with appropriate clinical protocols. Operational efficiency improvements occur through optimized resource utilization and a decrease in administrative burden.

### 9.2. Practical Implementation Recommendations

Healthcare organizations should implement the LPMDC framework through phased approaches, starting with mature technologies that have proven clinical benefits. Sepsis prediction algorithms with demonstrated mortality reduction benefits represent ideal initial implementations. Gradual expansion to respiratory and cardiovascular monitoring applications follows evidence-based progression.

Staff training programs should emphasize human–AI collaboration rather than AI replacing clinical judgment. Clinicians require an understanding of the strengths and limitations of AI systems to optimize the integration of decision-making. Continuous education ensures adaptation to evolving technology performance.

Infrastructure investment should prioritize improvements in interoperability and data quality before implementing advanced AI. Robust data governance frameworks ensure regulatory compliance and protect patient privacy. Cybersecurity measures must address increased connectivity and data-sharing requirements.

### 9.3. Economic and Healthcare System Impact

Economic considerations are crucial for practical adoption. Notably, many AI-based healthcare programs have demonstrated cost-effectiveness by improving outcomes and reducing waste. The findings of a prior systematic review reveal the capacity of AI tools to improve diagnostic accuracy and quality-adjusted life years, while reducing unnecessary procedures, resulting in a beneficial cost-effectiveness ratio. Namely, AI screening solutions may reduce the need for follow-up tests, resulting in system-wide savings. Nevertheless, the same research work warns that various published findings overlook real-world costs, including IT infrastructure, model training, and maintenance [189]. To overcome this issue, the LPMDC framework plan incorporates a thorough financial impact analysis. In addition to the initial model development and deployment, we will also consider the sustained costs, including data storage, computation (primarily for retraining or cloud inference), and user training. Additionally, we will assess the overall ownership cost in relation to hardware refresh cycles and software updates. Scalability is a key design consideration: through leveraging cloud-native services and container-based components, the system can be scaled progressively as patient data grows. Ultimately, our primary objective will be to demonstrate that LPMDC offers value for money by enhancing care efficiency while maintaining financial sustainability.

The integration of the LPMDC framework exhibits effectiveness in producing reliable returns on investment through enhancing patient outcomes and practical efficiency. Evidence from prior sepsis care and critical care quality improvement studies illustrates the breadth of potential benefits. For instance, large-scale hospital programs implementing early recognition and therapeutic protocols have documented relative mortality reductions of approximately 30–35% [195,196]. Systematic reviews of ICU and critical care pathway management have shown declines in length of stay for heart failure patients by approximately 1.9 days [197]. One ICU research study reports that implementing an automated sepsis alert reduced ICU length of stay by about 2.1 days [195]. Similarly, cohesive sepsis-bundle strategies have increased adherence, with clinical-bundle rates rising from 26.5% to 70%, resulting in notable improvements in outcomes, including a decline in 28-day mortality from approximately 50% to approximately 32%. In addition, bundle compliance was independently related to lower adjusted mortality, with an odds-ratio (OR) of 0.61 [196].

Reduced clinician cognitive load and improved workflow efficiency were implemented to address healthcare workforce shortages through enhanced productivity. Administrative burden reduction enables focus on direct patient care activities. Improvements in job satisfaction may reduce healthcare worker turnover and associated costs.

Future research should focus on prospective clinical validation through randomized controlled trials, long-term outcome assessment, and optimization of implementation strategies. Healthcare 5.0 systems and related technologies, including quantum computing, federated learning, and explainable AI, offer additional advancement opportunities.

The LPMDC framework represents significant progress toward realizing the full potential of AI in healthcare while maintaining the human elements essential for compassionate and ethical patient care. Successful implementation could transform critical care delivery through enhanced clinical decision-making, improved patient outcomes, and optimized resource utilization. Healthcare 5.0-enabled systematic AI integration offers practical solutions to current healthcare challenges while preparing systems for future medical advancement.

## Figures and Tables

**Table 1:** Sites and Validation Focus of the LPMDC.

Institution/Center	Service/Department	Validation Focus	Number of Theses/Dissertations
National Computer Center (CNI), Ministry of Health	Interoperability and Systems Department	Interoperability, technical integration, and AI deployment for clinical decision-making	9 Masters
National Center for Organ Transplantation (CNPTO), Tunis	Transplant Coordination	Data flow management, post-operative monitoring, traceability	4 Masters
Tunis Military Hospital	Intensive Care & Cardiology and a physician	Patient monitoring, AI for complication prediction	2 PhD, 2 Masters
Al Matri Hospital	Colorectal Surgery	Surgical simulation and optimization of operative protocols	2 Master
Memi University Hospital	Radiology	Medical imaging, PACS–AI integration	1 Masters
Mongi Slim University Hospital	Intensive Care and a physician	Real-time monitoring, post-pandemic solutions	1 PhD, 1 Master
Razi Hospital, Tunis	Neurology	Longitudinal follow-up, early detection of relapses	1 PhD, 2 Master’s
Cross-cutting Projects (Pandemic & Post-pandemic)	Various hospital services	Tele-monitoring of COVID and post-COVID patients, AI integration for continuity of care	1 PhD, 1 Master [18]

**Table 2:** Overview of Studies Included in the Review.

1st Author, Years	Type of Technology	Clinical Applications	Limitations Compared to LPMDC	Journals (Quartile)
Smith, J., 2022 [19]	Wearable sensors (skin, axilla) and invasive core probe	Optimizing neonatal thermal monitoring in the ICU to detect early temperature variations	-Focused solely on temperature monitoring in neonates -Lacks predictive AI/ML integration, multi-metric outcomes	Journal of Neonatal Nursing (Q2)
Geoffrey Chase, J., 2023 [20]	Digital twins and AI-based prediction detection support	Developing digital twins in medicine: automating cyber-physical-human systems to improve treatment in the ICUs	-High computational and data requirements for real-time simulation. -Integration with patient-specific dosimetry is limited	Cyber–Physical–Human Systems: Fundamentals and Applications (Q2)
Walinjkar, A., 2018 [21]	Wearable sensors kit and smart monitoring system	Using wearable sensors to monitor in real-time by predicting trauma scores (National Early Warning Score, Revised Trauma Score, Trauma Score-Injury Severity Score) and Predicting Survival, using physiological data	Limited to physiological parameters; does not integrate multimodal patient data or predictive modeling at the clinical decision support level	Applied System Innovation (Q1)
Wang, H., 2023 [22]	Contactless sensor, IoT-based monitoring	Using optical sensors for non-contact physiological assessment and early detection in a remote patient monitoring system using IoT-enabled CCTV cameras	-Requires complex data processing and network bandwidth -Less portable and not wearable; not suitable for low-power, personal medical devices	IEEE Internet of Things Journal (Q1)
Fragasso, T., 2011 [23]	Wearable sensors, contactless sensors and mHealth app	Validation and optimization of thermal monitoring methods in the neonatal ICUs to ensure accurate and reliable monitoring, early detection of anomalies, and adaptive temperature management	Limited integration with multi-parameter data collection may provide less comprehensive physiological monitoring; potential data gaps due to sensor placement or signal interference may require frequent calibration to maintain accuracy in neonatal ICU settings	Artificial Organs (Q2)
Rais-Bahrami, K., 2002 [24]	Wearable sensors and continuous blood gas monitoring sensors	Implementation of a precise and less invasive continuous blood gas monitoring approach for optimal assessment and early detection of imbalances in newborns in the ICUs	Limited long-term monitoring in extremely low birth weight infants Less flexible for integration with multiple physiological parameters compared to LPMDC	Journal of Perinatology (Q1)
Matey-Sanz, M., 2024 [25]	Smartphone, smartwatch, mHealth app, AI, wearable sensor	Developing mHealth systems using AI and sensors for predicting and detecting motor disorders (as part of remote care management strategies)	Limited precision in capturing complex motor patterns compared to lab-based or high-fidelity LPMDC systems Dependency on user compliance	IEEE Journal of Biomedical and Health Informatics (Q1)
Cheng, V. C., 2011 [26]	IoT-based monitoring + AI prediction decision support	MedSense combines automated monitoring, predictive analytics, and feedback to improve hand hygiene compliance in the ICUs	Limited to hand hygiene monitoring; does not integrate multi-source patient data or real-time personalized clinical decision support	BMC Infectious Diseases (Q1)
Cheng, S. M., 2021 [27]	Wearable sensors: wireless respiratory rate sensor	Integrating wireless sensors to monitor respiratory rate, detect, and prevent postoperative respiratory depression in gynecological intensive care	The short duration of monitoring meant that long-term outcomes and complications were not assessed	Indian journal of anaesthesia (Q2)
Young, A., 2013 [28]	IoT-based sensor/physiological monitoring devices	Personalizing hemodynamic treatment in the ICUs by predicting the response to vascular filling	Small sample size and limited patient diversity; only evaluated in controlled ICU settings; does not employ advanced machine learning models or continuous long-term monitoring	Journal of cardiothoracic and vascular anesthesia (Q2)
Gopalakrishnan, S., 2024 [29]	Wearable sensors, mHealth app	The STARS system automates urinary catheter monitoring in the ICUs and predicts infections	Requires wearable sensors and app infrastructure, which might limit scalability in low-resource settings Lacks flexibility in capturing multi-source patient data beyond urinary catheters	IEEE Transactions on Biomedical Engineering (Q1)
Li, Z., 2023 [30]	Contactless/wearable sensors	Detection and management of metabolic imbalances in the ICUs using passive smart lenses for real-time blood glucose monitoring	Limited validation in diverse ICU patient populations; performance under variable physiological conditions remains uncertain	Advanced Functional Materials (Q1)
Breteler, M. J., 2020 [31]	Wearable sensors	Predicting and detecting postoperative deterioration in the ICUs using wearable sensors	Limited generalizability due to a single-center study and a small sample size, which may not capture the full variability of patient populations	Anesthesiology (Q1)
Capp, N., 2019 [32]	Contactless/wearable sensors, AI-based monitoring	Predicting and detecting acute decompensation in chronic obstructive pulmonary disease/asthma patients by using intelligent respiratory monitoring	Limited generalizability due to a small and homogeneous patient cohort	IEEE Signal Processing in Medicine and Biology Symposium (SPMB) (Q2)
Chou, Y. A., 2023 [33]	IoT (smart sensor)	Smart IoT monitoring of air quality in the ICUs to detect occupancy-related CO_2_ spikes to optimize health safety	Limited generalizability due to the study being conducted in a single ICU setting with specific COVID-19 visitation restrictions, which may not reflect typical ICU conditions	Frontiers in Medicine (Q1)
Fries, J., 2012 [34]	Modeling, smart system, AI-assisted monitoring	Modeling caregiver flows to predict and optimize hand hygiene monitoring in the ICUs	Relies on human observation and modeling, which may introduce observer bias and lack the real-time automated monitoring capability present in LPMDC	Infection Control & Hospital Epidemiology (Q1)
Mariani, S., 2021 [35]	Telemonitoring, mHealth app	Telemonitoring of left ventricular assist device patients for the early prediction and detection of complications and treatment adjustment in the ICUs during the COVID-19 pandemic	Limited sample size and short follow-up period, which may affect the generalizability of the findings	Asaio Journal (Q1)
Ortiz-Barrios, M., 2023 [36]	AI/simulation	AI is used to analyze patient data from the emergency department to predict the likelihood of ICU admission. These predictions are integrated into a discrete event simulation model to observe ICU bed occupancy in real-time and identify current bottlenecks	This study relies on AI predictions integrated into a simulation without validating the model against real-time ICU admission outcomes, which may limit the generalizability and accuracy of its capacity management insights	Journal of Business Research (Q1)
Roncancio-Clavijo, A., 2023 [37]	AI predictive modeling	Predict disease severity and detect ICU patients at risk of clinical deterioration based on AI predictive models using blood test data	Limited generalizability due to the relatively small sample size and single-center data	PLOS One (Q1)
Di Napoli, A., 2023 [38]	Deep Learning–based Predictive Analytics	Predict mortality, intubation, and ICU admission based on deep learning algorithms using 3D chest CT images and clinical data	The model requires high-quality 3D CT scans and extensive clinical data, which may limit its generalizability to settings where such data are not readily available	Journal of Digital Imaging (Q2)
Ali, F. I., 2023 [39]	IoT (monitoring system)	IoT-based health monitoring system in the ICUs: monitoring of vital signs and prompt detection of clinical changes	Limited integration with predictive models for patient deterioration	International journal of online and biomedical engineering (Q2)
Sharma, S., 2023 [40]	Telemedicine/Remote Patient Monitoring Technology	Telemedicine in the ICUs	Focuses on general AI telemedicine challenges but lacks patient-specific predictive modeling	Journal of education and health promotion (Q2)
Guarrasi, V., 2023 [41]	AI	AI-based models are utilized in ICUs to predict disease progression, identify high-risk cases, and monitor patient status using chest X-rays and clinical data	Lack of comprehensive integration of multi-modal patient data beyond imaging and basic clinical metrics	Computers in Biology and Medicine (Q1)
Bartenschlager, C. C., 2023 [42]	Machine Learning for Clinical Prediction	AI can predict infection status and detect symptomatic COVID-19 cases using laboratory data	This study is limited by its focus on laboratory data	ACM Transactions on Management Information Systems (Q1)
Tasnim, N., 2023 [43]	Explainable Artificial Intelligence for clinical risk prediction	Predict mortality risk accurately and identify clinical risk factors using AI to optimize ICU resource allocation	This study is limited by its focus on specific datasets, which affect the generalizability of the AI model to other populations or clinical settings	Applied Sciences (Q2)
Kołodziejczak, M. M., 2023 [44]	AI	Predict patient deterioration by monitoring ongoing conditions in the ICUs using AI models	A conventional AI approach, lacking continuous feedback and corrective capabilities	Journal of Personalized Medicine (Q2)
Agrimi, E., 2023 [45]	AI-driven biomechanical simulation modeling	AI-based biomechanical simulations can predict respiratory function decline in the ICUs using lung CT scans and arterial blood gas data	AI-based biomechanical simulations without integrating the continuous monitoring and adaptive correction capabilities	The European Physical Journal Plus (Q2)
AlShehhi, A., 2024 [46]	ML	AI-based models help monitor disease progression and detect early signs of deterioration in ICU patients by using EHR	Limited by its retrospective design and reliance on EHR data, which restricts real-time applicability and comprehensive Healthcare 5.0 integration	PLOS One (Q1)
Genc, A. C., 2023 [47]	AI	AI models forecast mortality risk at very early stages in the ICUs, monitor patients in critical states, and recognize those at the highest risk	Narrower scope, lacking continuous monitoring and real-time corrective feedback for ICU patients	European Review for Medical & Pharmacological Sciences (Q2)
Charkoftaki, G., 2023 [48]	AI	Predict disease severity and monitor patient status in real-time in the ICU. Detection of key biomarkers associated with serious complications (decrease in serotonin levels) to identify patients requiring intensive care early	This study is limited by its reactive, ICU-focused approach, which lacks continuous monitoring and corrective feedback loop	Human Genomics (Q1)
Guevarra, K., 2025 [49]	AI-based imaging analysis	Prediction of clinical deterioration, monitoring of hemodynamic status, and complication detection in the ICUs by an AI-based model using imaging data	Focuses primarily on ICU imaging data and lacks integrated prediction, monitoring, detection, and correction	Current Cardiology Reports (Q1)
Niles, D., 2009 [50]	Continuous learning and skill-monitoring technology	Cardiopulmonary resuscitation training in the ICU is based on continuous learning, with monitoring and immediate correction of techniques, allowing for rapid and lasting mastery of skills	Traditional ICU training methods lack integration with constant, data-driven monitoring and corrective feedback	Resuscitation (Q1)

## Data Availability

The datasets supporting the conclusions are available from the corresponding author upon reasonable request.
